# Using Biomod2 and spatial quality modeling for comprehensive production zoning of *Bupleurum chinense* DC

**DOI:** 10.3389/fpls.2026.1915251

**Published:** 2026-09-04

**Authors:** Huihan Tang, Zhilin Gao, Huizhong Xu, Fanyun Meng

**Affiliations:** Engineering Research Center of Natural Medicine, Ministry of Education, Faculty of Geographical Science, Beijing Normal University, Beijing, China

**Keywords:** *B. chinense*, Biomod2, comprehensive production zoning, secondary metabolites, spatial quality

## Abstract

**Introduction:**

*Bupleurum chinense* DC. (*B. chinense*), an important traditional medicinal herb in China, exhibits several pharmacological activities, including antidepressant, anti-inflammatory, and hepatoprotective effects. Its medicinal value is highly dependent on the types and contents of secondary metabolites, such as saikosaponins. With the growing global emphasis on natural medicines in recent years, the market demand for *B. chinense* has been expanding, increasing the need for stable yield and high medicinal quality.

**Methods:**

This study constructed a comprehensive production zoning system by integrating ecological suitability modeling and spatial quality prediction. We utilized the Biomod2 platform to predict the distribution of ecological suitability and identify environmental factors affecting growth. Subsequently, a Principal Component Analysis - Multiple Linear Regression - Spatial Prediction framework was applied to evaluate spatial quality.

**Results:**

(1) The ensemble model constructed using the biomod2 platform (TSS = 0.851, AUC = 0.980) was employed to evaluate ecological suitability. The results indicated that the ecological suitability was higher in northern regions than in southern regions, with precipitation (Bio13, Bio17) and temperature (Bio6, Bio3, Bio2) were identified as the major driving factors. (2) Climatic factors (Bio15, Bio17) and soil factor (tp05) significantly affected saikosaponin content, while topographic factors exerted only a weak influence. Based on a regression model constructed via PCA-MLR-PC (R^2^ = 0.28, VIF< 5, Cook’s distance< 1 for 95% of observations), the relative content of the medicinal material was found to be higher in northern areas compared to southern areas. (3) The comprehensive production zoning of *B. chinense* showed that the high production zone was primarily observed in the Loess Plateau, the northern part of the North China Plain, and parts of the Northeast China Plain. The high production zone covered 1.1×10^6^km^2^; the medium production zone covered 0.7×10^6^km^2^, and the low production zone had the covered 0.4×10^6^km^2^.

**Discussion:**

The comprehensive framework that integrated both ecology and quality, representing the synergistic advantage between ecological suitability and medicinal quality. These findings distinguished different grades of production zones, providing a reference for prioritizing the development of high production zones and implementing differentiated cultivation strategies across various production zones.

## 1 Introduction

Bupleurum chinense DC. (*B. chinense*), a commonly used natural medicine, possesses broad medicinal value. *B. chinense* is widely distributed across China, encompassing Northeast China, North China, Northwest China, East China, and Central China [Tan et al., 2005]. Among these, provinces such as Shaanxi, Shanxi, and Hebei are recognized as core producing areas [Zhang et al., 2013]. Its wild resources are found on relatively dry slopes, forest edges, glades within forests, grasslands, and along roadsides. Existing literature has shown that the distribution and growth of *B. chinense* are affected by various ecological factors, including geographical position and climate [Qi et al., 2020]. In the past, medicinal *B. chinense* resources were mainly wild; however, with the traditional Chinese medicine industry expanding rapidly and market demand increasing owing to its significant medicinal value, wild *B. chinense* resources have experienced a sharp decline owing to overharvesting and ecological changes. In recent years, some regions have begun to adopt artificial cultivation of *B. chinense* [Lei et al., 2014]. However, traditional cultivation practices often overlook the profound influence of ecological conditions on *B. chinense*, affecting its yield and value. Therefore, understanding the environmental mechanisms driving the distribution and growth of *B. chinense* is crucial for refining planting strategies and facilitating the sustainable utilization and development of this valuable medicinal resource.

Research indicates that the major active components of medicinal herbs are secondary metabolites [Riaz et al., 2023]. In *B. chinense*, the primary secondary metabolites are saikosaponins, which have broad pharmacological activities, including antidepressant effects [Zhang et al., 2023], anti-inflammatory effects [Ma et al., 2025], and hepatoprotective effects [Chen et al., 2020]. As the recognition of traditional Chinese medicine continues to rise both domestically and internationally, the market demands higher standards for the quality and stability of medicinal materials. Modern plant physiology holds that moderate environmental stress can promote the accumulation of these metabolites [Huang and Guo, 2007], and their types and contents vary significantly under different stress conditions. Regarding *B. chinense*, abiotic factors—including light intensity, temperature variation, drought stress, and soil nutrients can influence the activity of key synthetic enzymes, which affect the synthesis and accumulation of major active components including saikosaponins [Deng et al., 2025], volatile oils, and flavonoids. Given the pivotal role of the ecological environment in governing metabolite synthesis, a systematic investigation into how these environmental variables affect secondary metabolites of *B. chinense* is crucial for optimizing medicinal quality and guiding the sustainable exploitation of this valuable resource.

Species distribution models (SDMs) serve as a core methodology in conservation biogeography, enabling the exploration of ecological niches and potential distributions based on known species distributions and environmental variables [Yin et al., 2013]. These models not only effectively analyze the relationship between environmental factors and species potential distributions, but also serve as a powerful tool for predicting changes in species distributions [Fourcade et al., 2018]. Currently, widely used species distribution models include Generalized Linear Model (GLM), Generalized Boosted Model (GBM), Artificial Neural Networks (ANN), Random Forest (RF), and Maximum Entropy Models (MaxEnt). However, single models exhibit certain limitations regarding predictive accuracy, stability, and generalization capability. Therefore, species distribution studies often employ ensemble modeling approaches. The Biomod2 platform encompasses a variety of commonly used species distribution models, and it can screen single models through strict evaluation criteria and construct ensemble models. It is currently a relatively mature species distribution modeling platform [Li et al., 2025].

Currently, research on *B. chinense* has predominantly focused on resource surveys and assessments [Jiang et al., 2009], determination of secondary metabolite content [Jiang and Chen, 2020], quality evaluation [Liu, 2024], and clinical pharmacology applications. Although some studies have already investigated the effects of environmental factors on *B. chinense* and its secondary metabolites, they often rely on single models or methods, failing to adequately integrate the synergistic mechanisms of ecology and quality. Consequently, a comprehensive production zoning system that simultaneously accounts for both ecological suitability for *B. chinense* growth and the environmental conditions favoring saikosaponin biosynthesis remains lacking. The study of medicinal materials should not only consider whether a species can survive but also whether it can efficiently synthesize secondary metabolites with medicinal value. To achieve the coordinated development of *B. chinense* resources regarding both yield and quality, it is necessary to move beyond the single-dimensional evaluation paradigm and construct a comprehensive system that considers both ecological suitability simulation and spatial quality prediction.

Therefore, this study utilized the Biomod2 platform for modeling, combined with correlation analysis and the Principal Component Analysis - Multiple Linear Regression - Spatial Prediction (PCA-MLR-SP) framework to construct an assessment system for comprehensive production zoning of *B. chinense*, and analyzed the major environmental driving factors.

## 2 Materials and methods

### 2.1 Research data

#### 2.1.1 Spatial data

Occurrence records of *B. chinense* were sourced from multiple databases, comprising the Global Biodiversity Information Facility (GBIF, http://www.gbif.org/), the Chinese Virtual Herbarium (CVH, http://www.cvh.ac.cn/) and relevant literature records concerning *B. chinense*. We acquired occurrence data for *B. chinense* using R, retrieving 835 records from multiple databases. To ensure data integrity, we retained only records with coordinate information and without obvious geospatial issues. Duplicate entries sharing identical latitude and longitude coordinates were subsequently removed, reducing the dataset to 386 unique occurrences. To further refine the data, we employed the CoordinateCleaner package to detect and eliminate spatially erroneous records, including those located in marine environments, national capitals, provincial centroids, and biological research institutions, and records with ambiguous taxonomic identification were also deleted. This rigorous filtering process yielded a dataset of 284 occurrence points. To address spatial autocorrelation, spatial thinning was performed using the spThin package in R with a minimum distance threshold of 20 km, retaining only one occurrence point within that threshold. This reduced the dataset to 160 valid records. Sample point data with secondary metabolites were also collected. We reviewed the relevant literature to collect and organize information on the content and distribution of secondary metabolites in *B. chinense*. Ultimately, data from 138 sample points of secondary metabolites were obtained.

**Figure 1:**
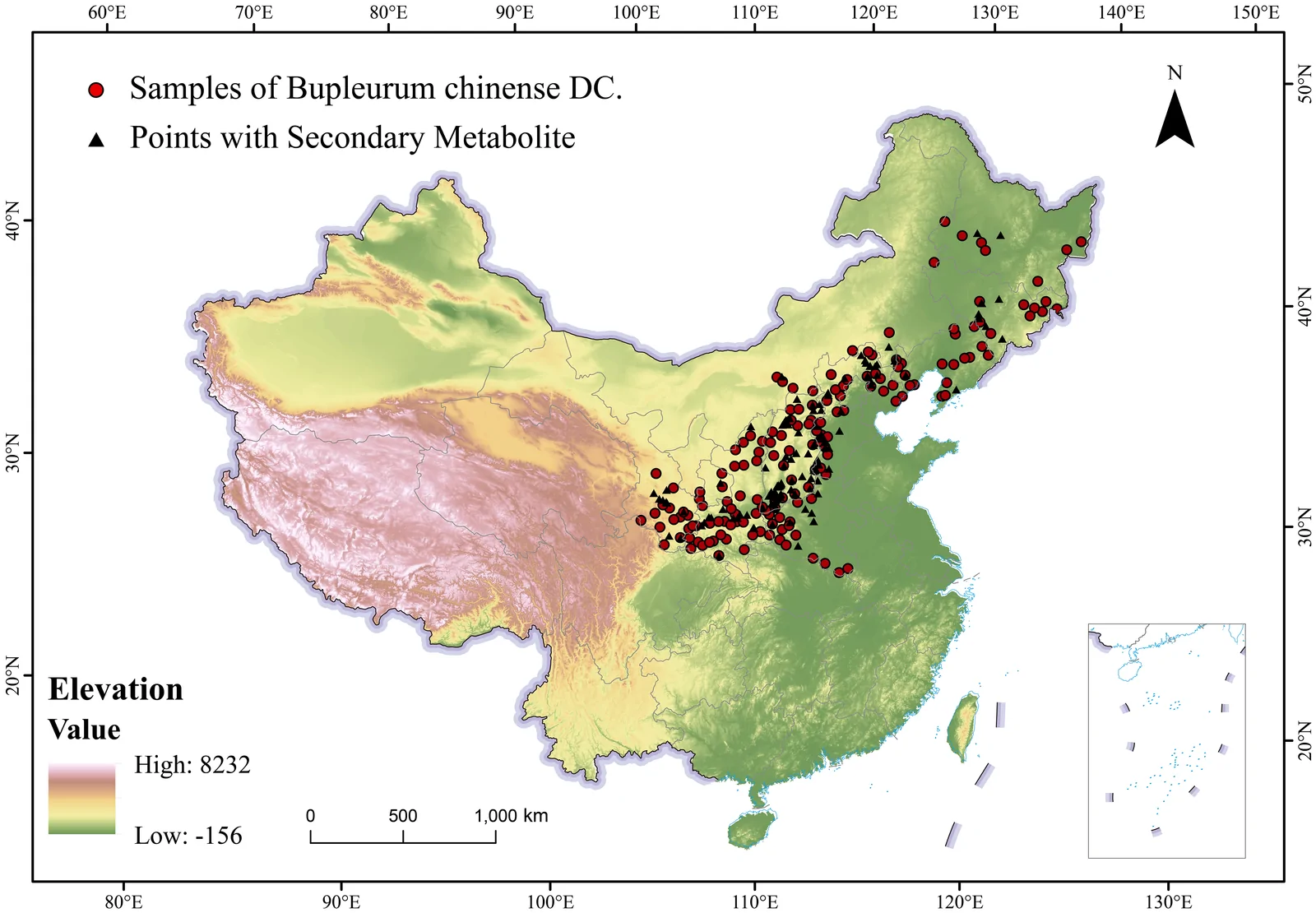
Spatial distribution of occurrence records and secondary metabolite sampling sites of *B. chinense.* (Map Approval Number: GS (2019)1835. Supervised by the Ministry of Natural Resources of the People’s Republic of China.).

To ensure the independence of the datasets used for ecological suitability modeling and secondary metabolite prediction, we conducted a spatial overlap analysis between the occurrence records and the sample points with secondary metabolites. The analysis revealed that only two sites share identical coordinates, accounting for approximately 1.25% of the 160 occurrence records. The mean distance between sampling sites and their nearest occurrence records is 35.32 km (median = 33.44 km), indicating substantial spatial separation. Due to the minimal extent of overlap and the different modeling objectives for the two datasets, these shared points were retained (**Figure 1**).

#### 2.1.2 Environmental factors

##### 2.1.2.1 Ecological suitability research data

For the purpose of improving the precision and trustworthiness of model and generating a result that aligns with reality, the selection of environmental variables should integrate the biological characteristics and habitat distribution features. In this study, we followed general guidelines for species distribution modeling and combined ecological characteristics and relevant research to select environmental factors. We identified three categories of environmental factors for ecological suitability research: Climate, soil, and topography.

Climatic factors: We selected 19 bioclimatic variables (Bio1-Bio19), including critical climate indicators. Additionally, solar radiation (srad) and vapor pressure (vapr) data were included to reflect light, heat, and atmospheric moisture conditions. All data represent the standard annual averages from 1970 to 2000 [Hijmans et al., 2005] and were sourced from the WorldClim (http://worldclim.org/). Soil factors: Soil datasets include China’s soil type data (soil), soil property data (clay1, clay2, sand1, sand2), and soil quality data (sq1-sq6) [Wei et al., 2012]. These data were obtained from the Harmonized World Soil Database (https://www.fao.org/soils-portal/). Topographic factors: These factors include elevation (elev), slope (slope), and aspect (aspect). Elevation values were extracted from the WorldClim (http://worldclim.org/), whereas slope and aspect were generated based on DEM data through the utilization of ArcGIS 10.8 (**Supplementary Table 1**).

To ensure spatial consistency, all environmental layers were resampled to a 30 arc-second resolution (approximately 1 km^2^). Strong multicollinearity among environmental factors can exacerbate model uncertainty, leading to redundancy and overfitting, which affects model accuracy. In this study, a Pearson correlation coefficient (|r| > 0.8) was defined as indicating strong collinearity [Chen et al., 2026]. Pearson correlation analysis was applied exclusively to continuous environmental variables (climatic and topographic factors) to identify collinear pairs. For those variable pairs exhibiting strong collinearity, we retained the variables with greater explanatory power based on the ecological habits and characteristics of *B. chinense*. For categorical soil factors, variable selection was instead based on prior research on the ecological preferences of *B. chinense*. Ultimately, 20 environmental factors were selected to enhance the robustness and ecological interpretability of the model (**Supplementary Table 2**, **Supplementary Figure 1** and **Supplementary Figure 2**).

##### 2.1.2.2 Spatial quality research data

In order to elucidate how environmental variables influence the synthesis and accumulation of major secondary metabolites in *B. chinense*, this study selected three categories of environmental factors, namely climate, soil, and topography (**Supplementary Table 3**), based on the comprehensive findings of existing studies [Zhang et al., 2025] and the properties of saikosaponins.

Climatic factors: In addition to the 21 environmental variables used in the ecological suitability study, three monthly climatic variables (tas5-10, pr5-10, SUNP5-10) and four derived bioclimatic variables (Bio20–Bio23) were included. The data were sourced from National Ecosystem Data Bank with a resolution of 1 km^2^ (https://www.scidb.cn/c/o00119/) [Hu et al., 2025]. Soil factors: This study incorporated soil nutrient and physicochemical property data at different soil depths (0-5cm, 0-20cm, 5-15cm, 15-30cm, 30-60cm, 60-100cm, 100-200cm), including soil organic carbon, pH, total nitrogen, total phosphorus, total potassium, cation exchange capacity, gravel content (>2mm), sand, silt, clay, soil texture type, bulk density, thickness, and other factors. All data were downloaded from the National Tibetan Plateau Data Center with a resolution of 1 km^2^ (http://data.tpdc.ac.cn/) [Liu et al., 2022, Liu et al., 2020]. Topographic factors: The same topographic factors selected for the ecological suitability study were used in this study.

### 2.2 Research methodology

**Figure 2:**
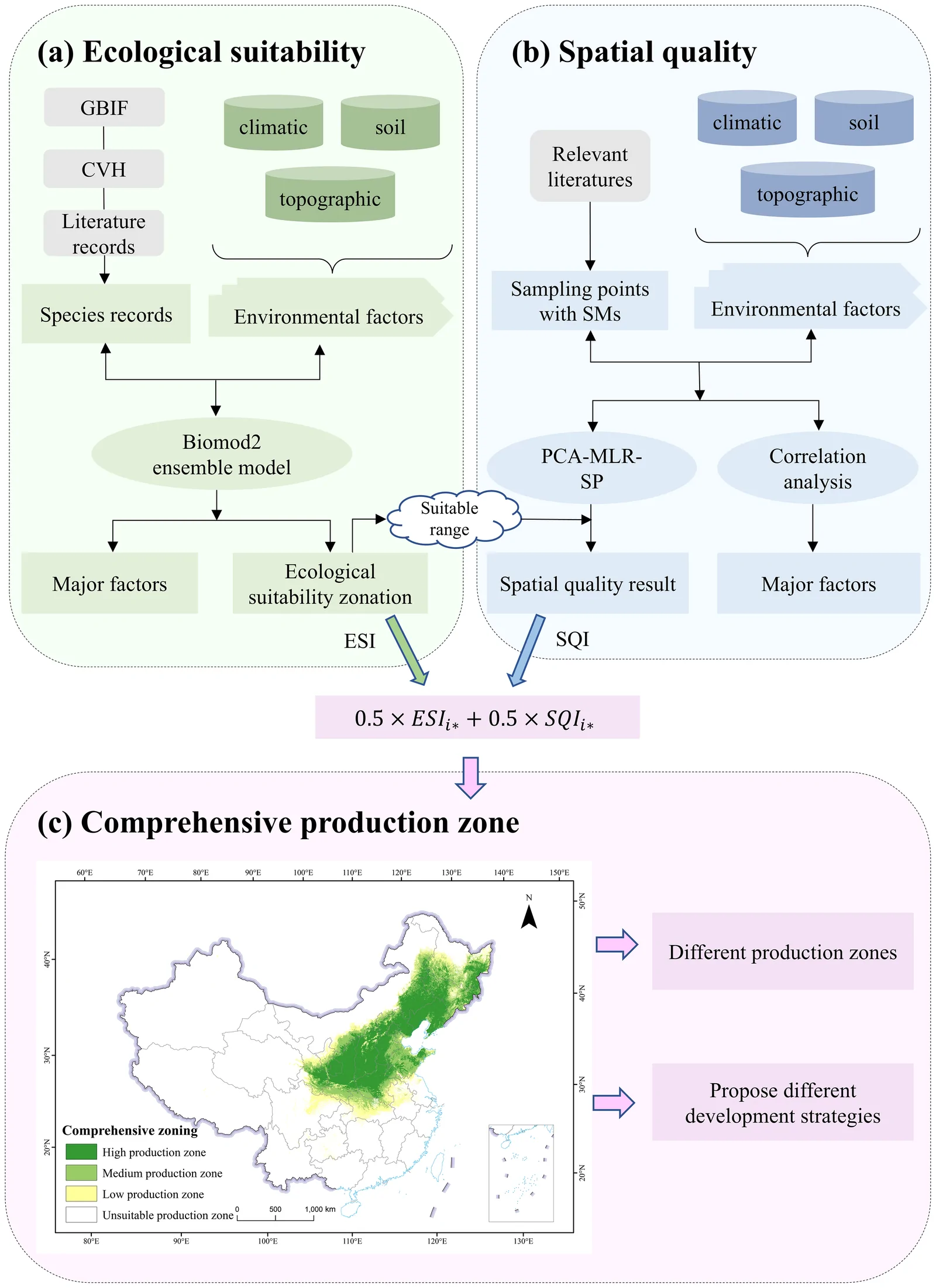
Technical process. **(a)** Ecological suitability; **(b)** Spatial quality; **(c)** Comprehensive production zone.

The comprehensive production zone study of *B. chinense* comprised ecological suitability modeling and spatial quality prediction (**Figure 2**).

#### 2.2.1 Ecological suitability research

This study employed the Biomod2 platform to run ten single species distribution models (GLM, GBM, GAM, CTA, ANN, SRE, FDA, RF, MARS, and MaxEnt). In the ensemble modeling phase, the performance of each single model was assessed based on evaluation metrics including Area Under the Receiver Operating Characteristic Curve (AUC), True Skill Statistic (TSS), and Cohen’s KAPPA (KAPPA) coefficient [Gama et al., 2016]. AUC serves as an indicator of discrimination capacity, with values varying between 0 and 1; higher scores denote enhanced predictive capability [Li et al., 2023]. The KAPPA coefficient evaluates model performance by measuring the agreement between the classification results and random guessing, making it a widely used metric for model validation. Its values range from -1 to 1, where higher values correspond to improved model precision. The calculation formula [Wang et al., 2007] is provided in Equation 1:

(1) $$KAPPA=\frac{\left(\frac{a+d}{n}\right)-\frac{\left(a+b\right)\left(a+c\right)+\left(c+d\right)\left(d+b\right)}{n^{2}}}{1-\frac{(a+b)\left(a+c\right)+\left(c+d\right)\left(d+b\right)}{n^{2}}}$$

In the equation, a represents true positives, referring to the number of units where the model correctly predicts the presence of a species; b represents false positives, indicating the number of units where the model incorrectly predicts species presence when it is actually absent; c represents false negatives, referring to the number of units where the model incorrectly predicts species absence when it is actually present; d represents true negatives, denoting the number of units where the model correctly predicts the absence of the species; n represents the total number of evaluation units in the confusion matrix.

TSS evaluates model performance by considering both sensitivity and specificity. Ranging from -1 to 1, higher TSS values signify greater predictive precision [Resquin et al., 2020]. The calculation formula is provided in Equation 2:

(2) $$TSS=sensitivity+specificity-1=\frac{ad-bc}{\left(a+c\right)\left(b+d\right)}$$

In the equation, a, b, c, and d are consistent with those in Equation 1.

In this study, we constructed ensemble models by selecting single models that performed well across evaluation metrics, using screening criteria of AUC≥0.80, and TSS≥0.75[Guo et al., 2025; Zhang et al., 2026]. In developing the ensemble model, the Committee Averaging (EMca) and the Weighted Average (EMwmean) methods were adopted to integrate the single models, aiming to mitigate the random errors inherent in single models through averaging. The EMca method does not differentiate between the performances of the single models and assigns equal weight to all selected models. The EMwmean method considers the predictive performance of each single model, assigning different weights to each model before calculating the weighted average.

To obtain the ecological suitability zones for *B. chinense*, we imported the ensemble model prediction results obtained from Biomod2 into ArcGIS 10.8. According to the suitability classification criteria, the ESI could be converted into species habitat suitability [Zhang et al., 2024]. Therefore, we classify the output into four levels: Ecologically unsuitable zone (0 - 0.2), low ecological suitability zone (0.2 - 0.4), medium ecological suitability zone (0.4 - 0.6), and high ecological suitability zone (0.6 - 1) [Yang et al., 2013].

#### 2.2.2 Spatial quality research

The quality indicators selected in this study included saikosaponin A, saikosaponin D, and total saikosaponins (the sum of saikosaponin A and D). The contents of saikosaponin A and D are quality indicators stipulated in the 2025 edition of the *Pharmacopoeia of the People’s Republic of China* that can reflect the quality of the medicinal material. By analyzing the effects of environmental factors on the contents of different saikosaponins, the differential regulatory mechanisms of these factors were elucidated. In this study, total saikosaponin content, which is more consistent with regulatory requirements and possesses more comprehensive application value, was used to construct a quality model.

In this study, environmental factors significantly correlated with saikosaponin content (p<0.05) were screened using correlation analysis. Based on the results, the key environmental factors exerting significant effects on saikosaponin content were identified. The PCA-MLR-SP framework is an integrated approach combining dimensionality reduction, regression modeling, and spatial prediction, designed to predict the spatial distribution of the relative saikosaponin content. Specifically, this study performed PCA separately on climatic and soil factors, applying the Kaiser criterion (eigenvalues > 1) to extract principal components. Subsequently, an MLR model was constructed using the extracted principal component scores as independent variables and the total saikosaponin content as dependent variable. Finally, the regression equation was applied to the low, medium, and high ecological suitability zones, excluding ecologically unsuitable zones to avoid interference with quality prediction. This process ultimately yielded the SQI representing the relative saikosaponin content, and a spatial quality map.

#### 2.2.3 Ecological quality research and comprehensive production zoning

Given the absence of empirical evidence favoring a specific contribution ratio between ecological suitability and quality, we adopted a neutral equal-weight scheme (0.5:0.5) as a baseline for integrating the two indicators. To verify the robustness of this baseline, a systematic weight sensitivity analysis was conducted (**Supplementary Figure 3**). The results showed that the Spearman rank correlation between the equal-weight EQI and EQI values derived from ESI weights ranging from 0.2 to 0.8 consistently exceeded 0.96, with most values above 0.99. This indicated that the spatial ranking of EQI was highly robust to weight variations across a broad range of weighting schemes. These results demonstrate that the 0.5:0.5 scheme falls within a robust region, providing a stable and reliable assessment of comprehensive production zones.

Both ESI and SQI were separately normalized to a [0, 1] range using min-max normalization to eliminate dimensional differences and ensure comparability. Following normalization, the EQI was calculated by performing a weighted overlay of the ESI and the SQI, as formulated in Equation 3:

(3) $$EQI_{i}=0.5\times ESI_{i*}+0.5\times SQI_{i*}$$

In the equation, $ESI_{i*}$ represents the normalized ecological suitability index of the unit; $SQI_{i*}$ represents the normalized spatial quality index of the unit; *EQI_i_* represents the ecological quality index.

Based on the EQI, the results were categorized into three classes: low production zone (0-0.4), medium production zone (0.4-0.7) and high production zone (0.7-1). The remaining ecologically unsuitable zones, where no quality prediction was conducted, were designated as unsuitable production zones, thus generating the comprehensive production zoning map of *B. chinense*.

## 3 Results

### 3.1 Ecological suitability study results based on Biomod2

#### 3.1.1 Model accuracy evaluation

**Figure 3:**
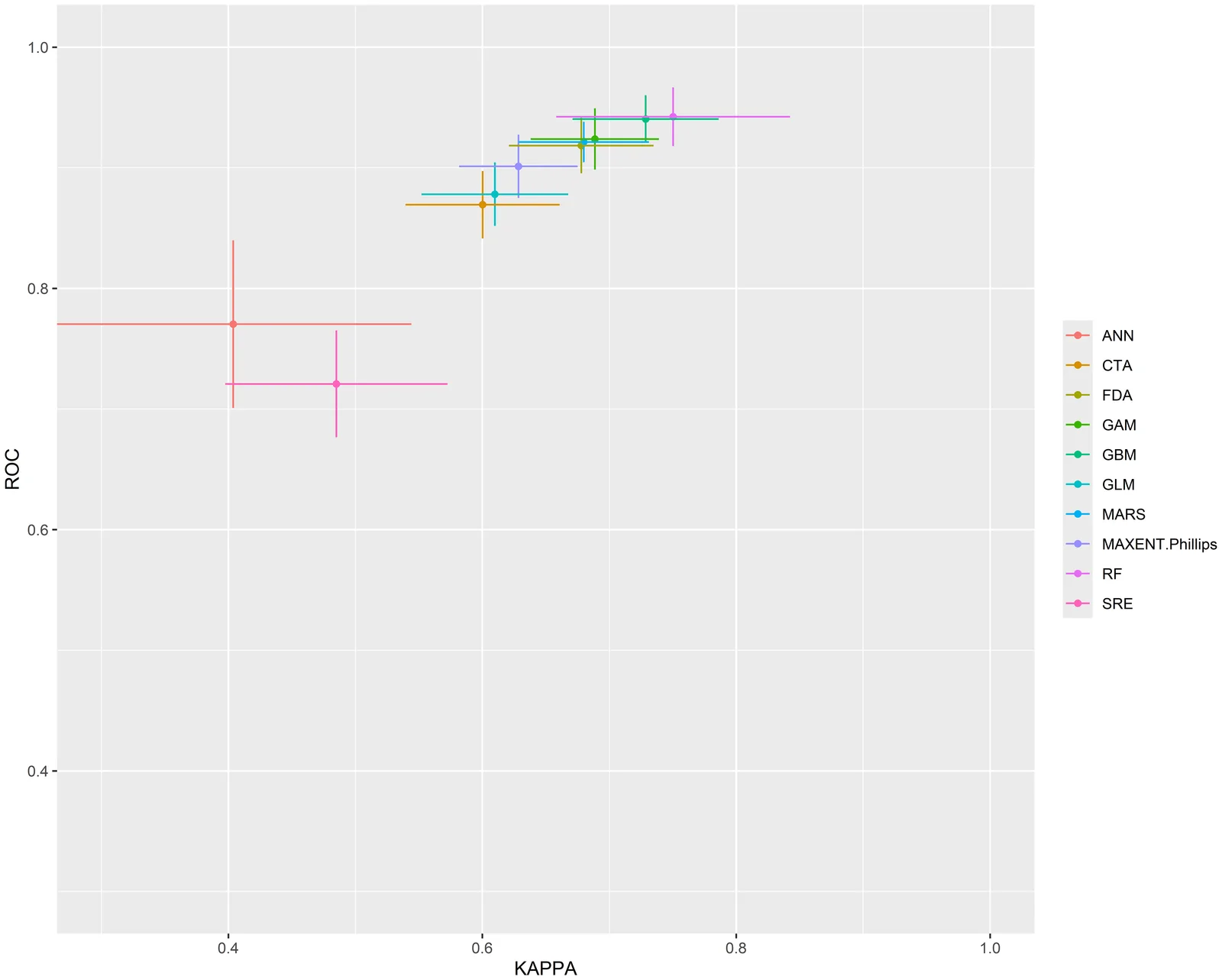
Accuracy evaluation results of single models.

This study utilized the Biomod2 platform to run ten single species distribution models. To evaluate their stability and reliability, we calculated key performance indicators for each single model, as shown in **Figure 3**. The majority of these models exhibited KAPPA coefficients greater than 0.6 and AUC(ROC) values exceeding 0.8, which indicated good predictive performance. The ANN and SRE models showed relatively lower predictive performance compared to the remaining models.

Utilizing the models that met the specified criteria, we constructed four different ensemble models using the EMca and the EMwmean methods (i.e., EMwmean by TSS, EMca by TSS, EMwmean by AUC, and EMca by AUC), and compared their performances (**Table 1**). Overall, all ensemble models demonstrated a high level of performance across the evaluation metrics. Furthermore, ensemble models constructed using the two methods generally outperformed the single models in terms of predictive accuracy and robustness. For TSS metric, all ensemble models scored above 0.82. Regarding the AUC values, all ensemble models scored between 0.96 and 0.98. For KAPPA metric, all ensemble models scored above 0.70, indicating high spatial predictive ability.

**Table 1:** Accuracy evaluation results of ensemble models.

Model	Metric	Value	Sensitivity (%)	Specificity (%)
EMcaByTSS	TSS	0.851	96.667	88.442
	AUC	0.980	96.667	88.442
	KAPPA	0.774	75.000	97.990
EMwmeanByTSS	TSS	0.844	96.667	87.538
	AUC	0.973	96.667	87.940
	KAPPA	0.738	81.667	94.874
EMcaByAUC	TSS	0.831	95.000	88.040
	AUC	0.972	95.000	88.342
	KAPPA	0.733	69.444	97.990
EMwmeanByAUC	TSS	0.828	96.111	86.734
	AUC	0.966	95.556	87.337
	KAPPA	0.705	73.889	95.779

#### 3.1.2 Ecological suitability results

Based on evaluation metrics, we selected the most appropriate ensemble model (EMcaByTSS) for predicting the distribution of ecological suitability for *B. chinense*. This selection was based on its superior performance across all evaluation metrics compared to other ensemble models. Specifically, the EMcaByTSS model achieved the highest TSS value (0.851) and the highest AUC value (0.980), indicating excellent discriminatory power between suitable and unsuitable habitats. Furthermore, this model exhibited the highest specificity (88.44%). High specificity is vital for this study as it represents the reliable exclusion of unsuitable habitats. Given its optimal balance of sensitivity (96.67%) and specificity, EMcaByTSS model was determined to be the most robust model for predicting the ecological suitability zones of *B. chinense*.

Using ArcGIS 10.8, the raster data from the ensemble model predictions were projected to the Lambert Conformal Conic (LCC) coordinate system, with the China Geodetic Coordinate System 2000 (CGCS2000) as the datum, the central meridian at 105°E, standard parallels at 25°N and 47°N, latitude of origin at 0°, and the linear unit in meters. This projection was selected for its conformality over mid-latitude regions, ensuring shape fidelity and angular preservation across the national-scale study area.

**Figure 4:**
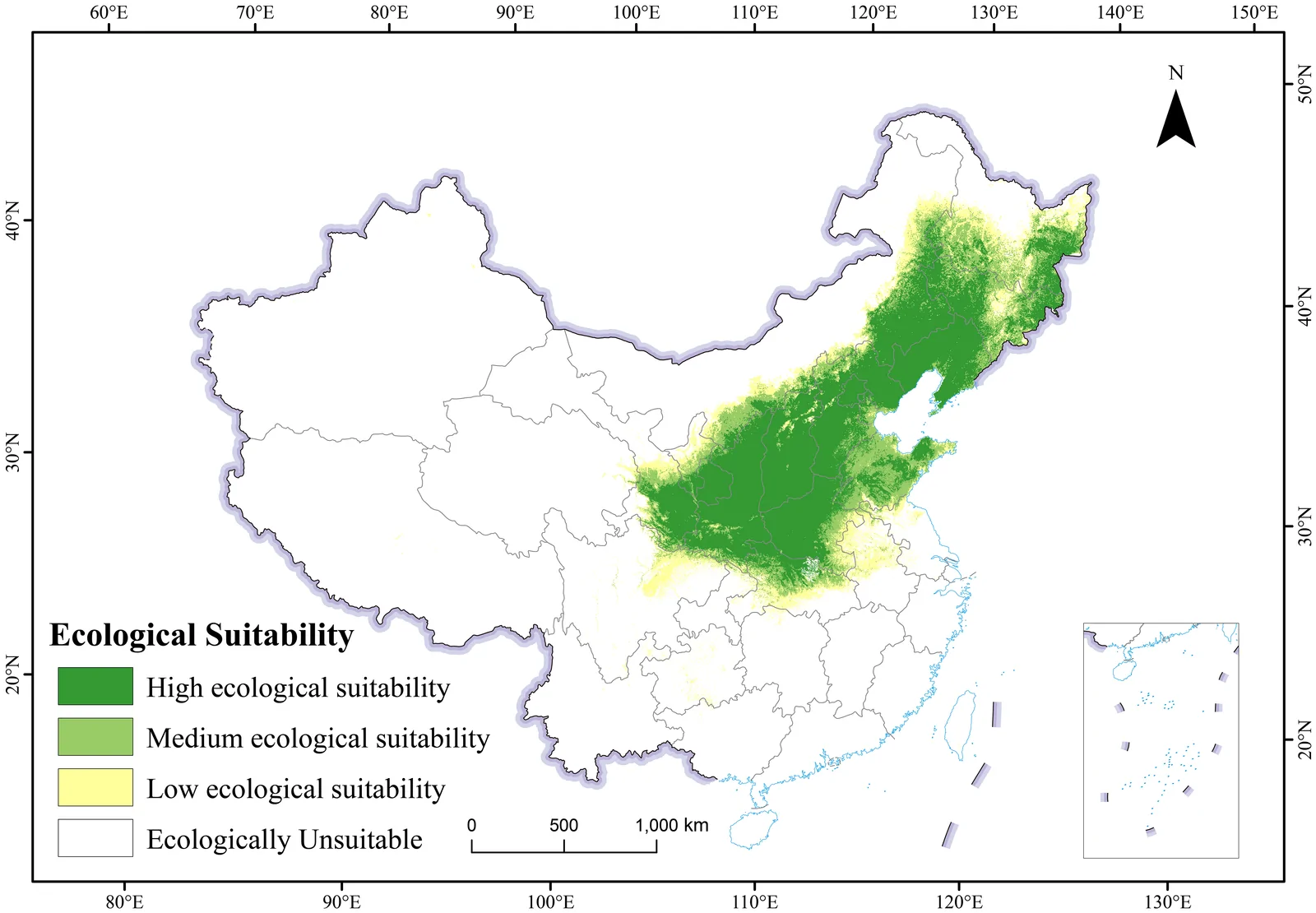
Ecological suitability results of *B. chinense.* (Map Approval Number: GS (2019)1835. Supervised by the Ministry of Natural Resources of the People’s Republic of China.).

The results of the ecological suitability revealed distinct regional variations and effectively delineated the ecological suitability zones of *B. chinense* across China **(Figure 4**). Spatially, suitability was generally higher in the northern regions compared to the southern regions, forming a gradient extending from the northeast to the southwest. The high ecological suitability zone was mainly distributed across the northern North China Plain, eastern Loess Plateau, and southern Northeast China Plain, covering Shanxi, Shaanxi, southeastern Inner Mongolia, Hebei, Liaoning, and Henan. This zone represented the core agglomeration area for *B. chinense* ecological suitability, where the natural environment aligned most closely with its growth requirements. The medium ecological suitability zone primarily extending around the periphery of the high ecological suitability zone, including southern Inner Mongolia, southwestern Heilongjiang, southern Jilin, and most of Shandong. The low ecological suitability zone scattered along the edges of the medium ecological suitability zone, such as northern Northeast China Plain, parts of the North China Plain, and the western edge of the Loess Plateau. Ecologically unsuitable zone extensively covered the northwest arid and semi-arid regions, the Tibetan Plateau, and most provinces of southern China.

According to the ensemble model results, the high ecological suitability zone for *B. chinense* covered an area of 1.3×10^6^ km^2^ (accounting for 13%), the medium ecological suitability zone covered an area of 0.5×10^6^ km^2^(accounting for 6%), the low ecological suitability zone covered an area of 0.4×10^6^ km^2^(accounting for 4%), and the ecologically unsuitable zone covered an area of 7.4×10^6^ km^2^(accounting for 77%). Among them, within the high ecological suitability zone, the provinces of Shaanxi (1.8×10^5^ km^2^), Inner Mongolia (1.6×10^5^ km^2^), Hebei (1.5×10^5^ km^2^), Shanxi (1.5×10^5^ km^2^), Liaoning (1.3×10^5^ km^2^), Henan (1.2×10^5^ km^2^), Jilin (1.0×10^5^ km^2^), and Gansu (0.9×10^5^ km^2^) accounted for relatively large areas.

### 3.2 Spatial quality study results based on correlation analysis and the PCA-MLR-SP framework

#### 3.2.1 Correlation analysis

As shown in **Table 2**, both the precipitation during the driest quarter (Bio17) and the coldest quarter (Bio19) exhibited significant positive correlations with saikosaponin A (r = 0.269, P< 0.01); conversely, mean diurnal temperature range (Bio2) and precipitation of the driest month (Bio14) displayed significant negative correlation (r = -0.254, P< 0.01). The content of saikosaponin D was significantly correlated with seasonal fluctuations in temperature and precipitation. Precipitation seasonality (Bio15) exhibited the most pronounced negative correlation (r = -0.334, P< 0.01), while temperature seasonality (Bio4) showed a significant negative correlation (r = -0.276, P< 0.01). The total saikosaponins showed significant negative correlations with precipitation seasonality (Bio15), temperature annual range (Bio7), mean diurnal temperature range (Bio2), the average percentage of sunshine during the growth period (SUNP5-10), and temperature seasonality (Bio4). In contrast, it exhibited significant positive correlations with precipitation of the driest quarter (Bio17) and precipitation of the coldest quarter (Bio19).

**Table 2:** Pearson correlation coefficients between saikosaponin content and climatic factors.

Climatic factor	A(r)	D(r)	Total(r)
Bio7	-0.164	-0.221**	-0.217*
Bio4	-0.170*	-0.276**	-0.255**
Bio13	-0.022	-0.234**	-0.160
Bio14	-0.254**	0.137	0.205*
Bio15	-0.212*	-0.334**	-0.311**
Bio17	0.269**	0.184*	0.242**
Bio2	-0.259**	-0.228**	-0.265**
Bio19	0.269**	0.184*	0.242**
Bio18	0.022	-0.170*	-0.098
Bio22	0.188*	0.226**	0.231**
SUNP5-10	-0.246**	-0.235**	-0.263**

(* indicates statistical significance at p< 0.05 level; ** indicates statistical significance at p< 0.01 level.)

As shown in **Table 3** and **Supplementary Table 4**, the physical structure and chemical properties of the soil were closely related to saikosaponin content. Among the physical structural indicators, soil texture was significantly correlated with saikosaponin content. Specifically, the silt content (btslt) showed a significant or extremely significant positive correlation with both saikosaponin D and total saikosaponins at multiple soil depths, while the sand content (btsnd) exhibited a significant negative correlation. Additionally, soil layer thickness (thickness) was positively correlated with saikosaponin content. In terms of chemical properties, soil pH and cation exchange capacity (cec) showed notable correlations with saikosaponin content. The pH values at various soil depths exhibited a positive correlation with saikosaponins, whereas cec showed significant or highly significant negative correlations with saikosaponin D and total saikosaponins. Furthermore, the availability of phosphorus and potassium in the soil also correlated with saikosaponins. Specifically, the total phosphorus content in the topsoil (tp05) showed a highly significant positive correlation with saikosaponins, and both total phosphorus density (tpd) and total potassium density (tkd) at multiple soil depths exhibited significant positive correlations with saikosaponin D.

**Table 3:** Pearson correlation coefficients between saikosaponin content and soil factors.

Soil factor	A(r)	D(r)	Total(r)
tp05	0.222**	0.328**	0.312**
tp515	0.14	0.300**	0.257**
tp1530	0.012	0.195*	0.130
tp3060	0.042	0.180*	0.134
tp100200	-0.187*	-0.027	-0.103
cec05	-0.055	-0.207*	-0.157
cec515	-0.077	-0.237**	-0.187*
cec1530	-0.072	-0.227**	-0.178*
cec3060	-0.108	-0.253**	-0.211*
cec60100	-0.116	-0.276**	-0.230**
cec100200	-0.090	-0.285**	-0.223**

(* indicates statistical significance at p< 0.05 level; ** indicates statistical significance at p< 0.01 level.).

As shown in **Table 4**, elevation (elev) showed a significant positive correlation with the content of saikosaponin D (r = 0.209, P< 0.05), and total saikosaponins exhibited a significantly negatively correlated with slope (r = -0.172, P< 0.05).

**Table 4:** Pearson correlation coefficients between saikosaponin content and topographic factors.

Topographic factor	A(r)	D(r)	Total(r)
slope	-0.161	-0.152	-0.172*
elev	0.002	0.209*	0.135

(* indicates statistical significance at p< 0.05 level; ** indicates statistical significance at p< 0.01 level..)

#### 3.2.2 Results of spatial quality study

Based on the correlation analysis, this study selected climatic and soil factors for PCA. The results were as follows: Two common factors were extracted from the climatic factors, with a cumulative variance contribution rate of 89.534% (**Supplementary Table 5**), while four common factors were extracted from the soil factors, with a cumulative variance contribution rate of 92.385% (**Supplementary Table 6**). The cumulative variance explained by the principal components approached or exceeded 90%, indicating that the extracted variables were highly representative and adequately summarized the information structure of the original factors. The extracted climatic and soil principal components were used as independent variables to construct the final multivariate linear regression equation for predicting the spatial quality of *B. chinense*, expressed as Equation 4:

(4) $$Y=0.686-0.075x_{1}+0.035x_{2}+0.055x_{3}-0.024x_{4}-0.015x_{5}+0.026x_{6}$$

In this equation, Y represents the relative content of saikosaponins, *x*_1_ represents ClimatePC1, *x*_2_ represents ClimatePC2, *x*_3_ represents SoilPC1, *x*_4_ represents SoilPC2, *x*_5_ represents SoilPC3, and *x*_6_ represents SoilPC4. (R^2^ = 0.28, adjusted R^2^ = 0.23, F = 3.357, p = 0.004).

**Figure 5:**
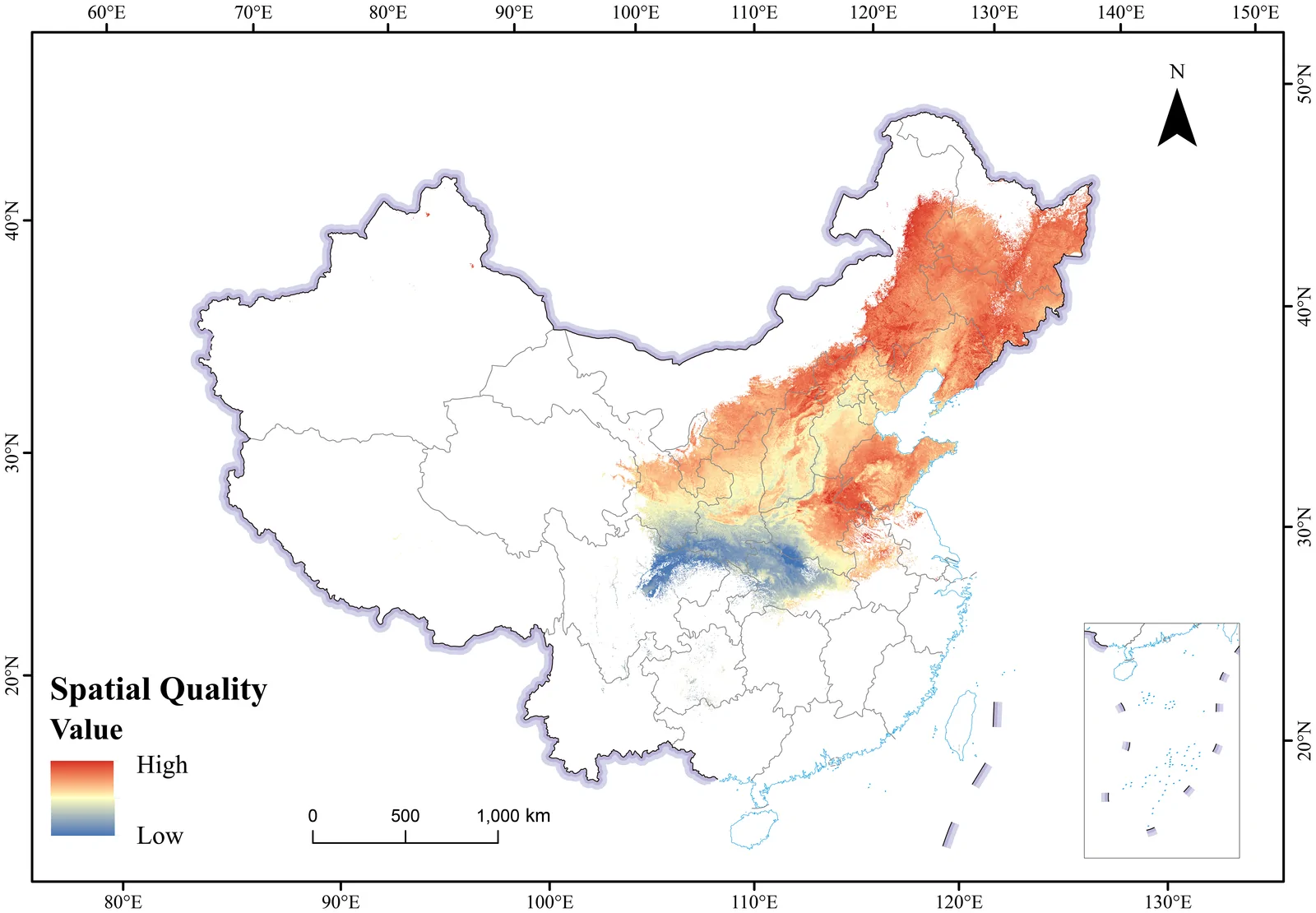
Spatial quality map of *B. chinense.* (Map Approval Number: GS (2019)1835. Supervised by the Ministry of Natural Resources of the People’s Republic of China.).

Based on the result, the SQI was calculated using ArcGIS 10.8. This index was applied to the high, medium, and low ecological suitability zones of *B. chinense* to generate a spatial distribution map of relative saikosaponin content, as shown in **Figure 5**. Overall, the spatial quality of *B. chinense* exhibited a distinct spatial gradient across China. The overall pattern demonstrated a clear trend of high in the east and low in the west, and high in the north and low in the south. The high-value zones were mainly concentrated in Heilongjiang, Jilin, Liaoning, eastern and southern Inner Mongolia, central and northern Shanxi, central and northern Shaanxi, and central Gansu. The medium-value zones were primarily distributed in western Hebei, central Henan, and central Shaanxi. The low-value zones mainly covered Hubei Province and northeastern Sichuan.

### 3.3 Comprehensive production zoning results

**Figure 6:**
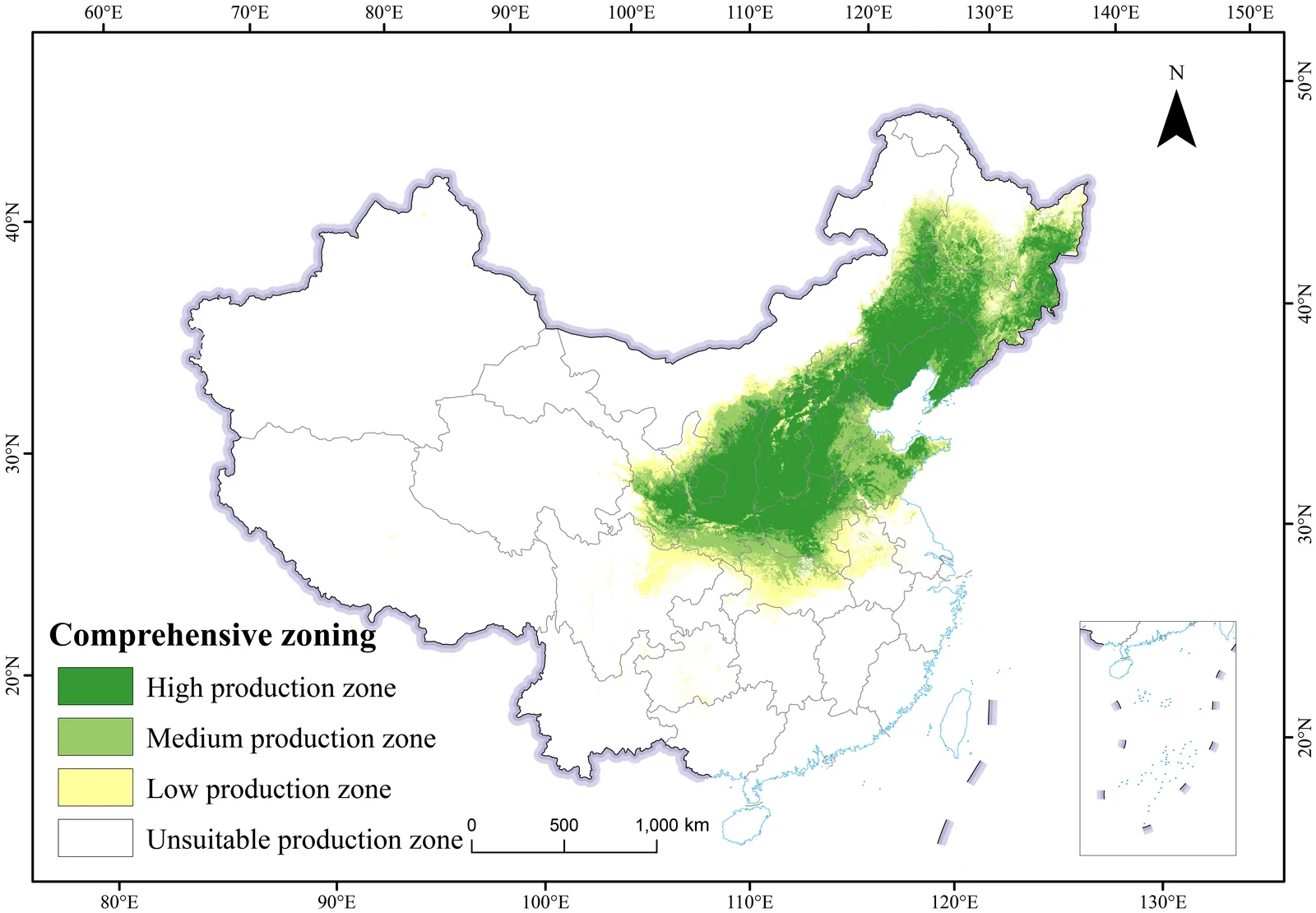
Comprehensive production zonation of *B. chinense.* (Map Approval Number: GS (2019)1835. Supervised by the Ministry of Natural Resources of the People’s Republic of China.).

The results (**Figure 6**) indicated that the comprehensive production zones of *B. chinense* exhibited distinct spatial differentiation and aggregation patterns, with an overall trend of higher grades in the north and lower grades in the south. The distribution of different production zones is jointly driven by natural geographical conditions, ecological environmental adaptability, and other factors, showing varied distribution characteristics.

In terms of spatial distribution, the high production zone was mainly concentrated in most parts of Shanxi, northern and central Shaanxi, southeastern Gansu, northern and eastern Hebei, southeastern Inner Mongolia, most of Liaoning, and western Jilin. The medium production zone extended outward from the core high production zone, covering southern Inner Mongolia, southern Gansu, southern Shaanxi, southern Henan, most of Shandong, southern Heilongjiang, and central Jilin. The low production zone was distributed around the periphery of the medium production zone, forming a distinct transition belt, mainly located in northeastern Sichuan, northern Hubei, and central Anhui. The unsuitable production zone was the most widely distributed, covering the northwest arid and semi-arid regions, the Tibetan Plateau, and most provinces of southern China.

In terms of area, among the three production zones, the high production zone had the largest area, covering 1.1×10^6^km^2^ (accounting for 11% of the total national area); the medium production zone covered 0.7×10^6^km^2^ (accounting for 7% of the total national area), and the low production zone had the smallest area, covering 0.4×10^6^km^2^ (accounting for 5% of the total national area).

## 4 Discussion

### 4.1 Model accuracy and ensemble model construction

The differential performance of single models stems from their algorithmic mechanisms, which directly determine their capabilities in data processing, relationship fitting, and disturbance resistance [Hallgren et al., 2019]. RF can capture the nonlinear relationships and complex interactions between environmental factors and species distribution, thereby achieving high accuracy and predictive capability in species distribution modeling [Zhang et al., 2019]. In contrast, although ANN can similarly simulate complex nonlinear relationships, they often exhibit unstable performance in species distribution modeling due to convergence difficulties or insufficient generalization ability, resulting in low predictive accuracy or overfitting. The SRE model fails to capture the complex relationships between environmental factors and species distribution; its predictions are directly output as binary outputs that only indicate the presence or absence of the species [Sang, 2015], unable to express the continuous gradient nature of ecological suitability. Consequently, due to their algorithmic assumptions and structural constraints, single models employ different identification and processing strategies for the same dataset, leading to significant variability in predictive performance.

Ensemble models constructed by integrating single models that meet predefined accuracy thresholds often significantly outperform single models in both predictive accuracy and robustness. This is achieved by synergistically leveraging the strengths of single algorithms and mitigating their inherent uncertainties. In recent years, various studies on the suitability of medicinal plants such as *Bidens biternata* [Liu et al., 2026], *Spatholobus suberectus* [Lin et al., 2025], *Aconitum pendulum Busch* [Wan et al., 2025], and *Epimedium acuminatum* [Wang et al., 2025] have all adopted the Biomod2 ensemble modeling. These studies have confirmed the reliability and significant advantages of the ensemble method in predicting the suitable zone of Chinese medicinal herbs.

### 4.2 Effects of environmental factors on the ecological suitability of *B. chinense*

**Figure 7:**
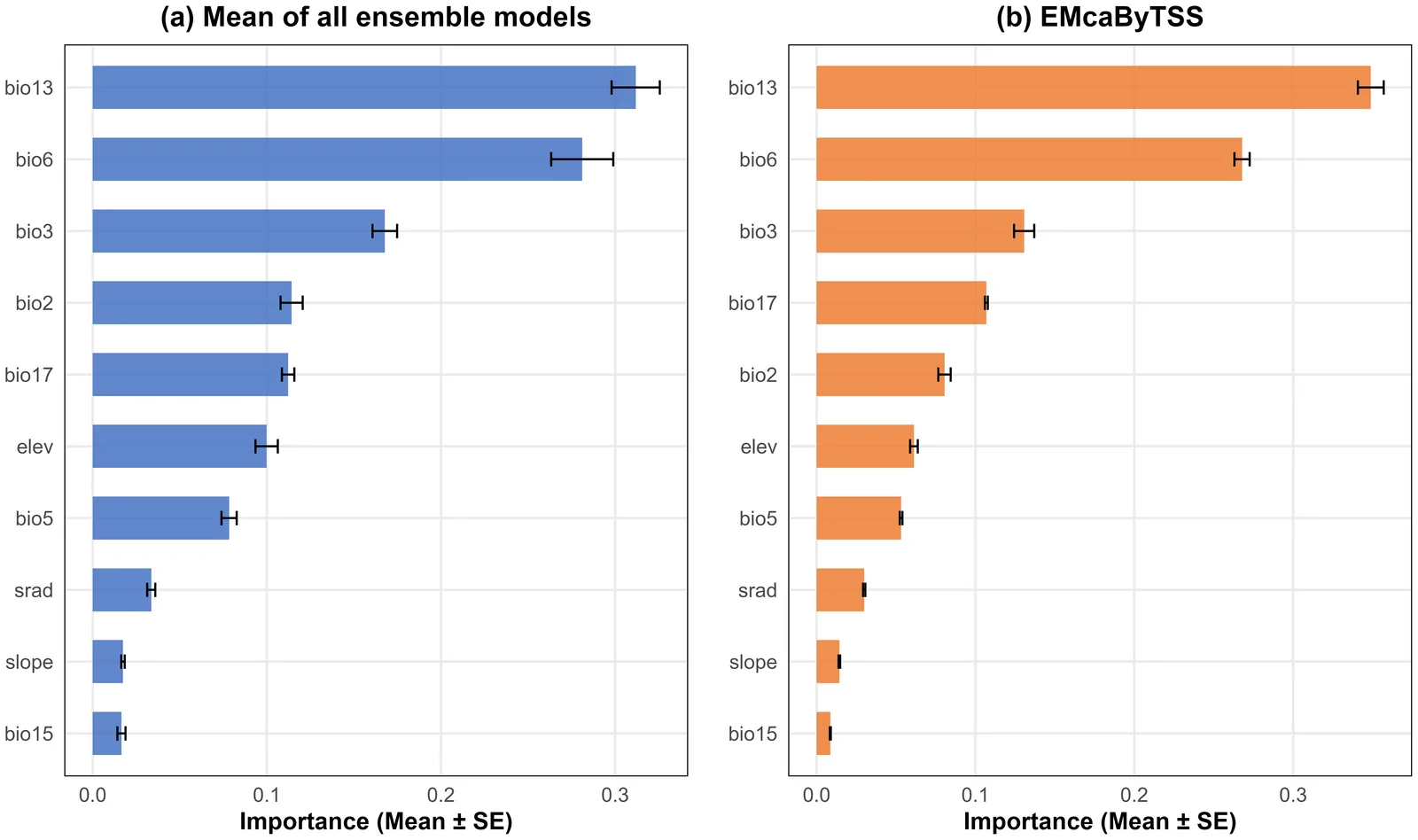
Contribution of major environmental factors. **(a)** Mean major environmental contribution rates across all ensemble models. **(b)** Major environmental contribution rates in the EMcaByTSS model.

The environmental drivers analysis revealed (**Figure 7**) that precipitation was the dominant factor governing the distribution of *B. chinense*, with Bio13 exhibiting the highest contribution rate. This hydrological dominance confirmed the important role of precipitation in seed germination and plant growth, and was highly correlated with the species’ core distribution in China’s temperate monsoon zone. Bio17 also exhibited a relatively high contribution rate, reflecting the species’ sensitivity to dry-season water availability. Although *B. chinense* possesses drought-tolerant characteristics, insufficient precipitation during the dry season can still induce cell dehydration, membrane system damage, enzymatic dysfunction, and metabolic disorders. Previous experiments have demonstrated that both extreme drought and excessive humidity can alter photosynthetic efficiency and biomass allocation [Liu and Zhou, 2022], thereby limiting the growth performance of this species within its hydrological niche. Temperature factors (Bio2 and Bio3) also have a high contribution rate in the model, likely acting through their influence on the balance between daytime photosynthetic carbon uptake and nighttime respiratory consumption. Genome-wide association analysis by Song et al. [Song et al., 2026] revealed that Bio3 is the environmental factor most strongly associated with genomic variation in *B. chinense*, further confirming the importance of Bio3 for this species. This reflects the important role of climatic factors, primarily precipitation and temperature, in shaping the ecological niche of *B. chinense*. These factors determine its macro-scale distribution, while topographic factors exerted influence on distribution patterns at regional scales.

Building on these ecological findings, we further compared our outputs with previous zoning studies. The environmental contribution rates obtained in this study are generally consistent with those of Chen et al. [Chen et al., 2022], which also confirmed Bio13 as the dominant environmental factor influencing the distribution of *B. chinense*. However, a notable difference was observed in the ranking of elevation contribution rates, reflecting methodological distinctions between the single-algorithm MaxEnt approach employed by Chen et al. and the ensemble modeling framework adopted herein. In our ensemble model, when climatic factors exert a dominant role, the apparent effect of a single topographic factor may be diluted. Meanwhile, the ecological suitability zones for *B. chinense* obtained in this study are highly consistent with previous studies [Wei et al., 2005]. The results are of reference value for guiding conservation prioritization and cultivation planning of *B. chinense*.

### 4.3 Effects of environmental factors on the spatial quality of *B. chinense*

Environmental factors exerted differential effects on the contents of various saikosaponin components, which may arise from the divergent pathways and magnitudes of environmental influences [Yang et al., 2026]. This finding provides valuable guidance for the extraction and utilization of medicinal materials based on specific saikosaponin types.

In terms of climatic factors, moderate drought stress can enhance the biosynthesis of secondary metabolites, whereas severe drought limits photosynthetic carbon fixation and depletes the precursor pools required for secondary metabolism [Selmar and Kleinwächter, 2013b]. Our result indicates that moderate precipitation during the driest quarter favors saikosaponin A accumulation. This finding suggests that an adequate water supply prevents extreme drought-induced metabolic inhibition and sustains the physiological basis for secondary metabolite synthesis, consistent with the finding that only moderate, not excessive, water stress is beneficial for natural product accumulation [Selmar and Kleinwächter, 2013a]. Conversely, high precipitation seasonality may exceed the adaptive threshold of the plant, thereby suppressing sustained saikosaponin accumulation [Yang et al., 2019]. Regarding soil factors, tp05 exerted a positive effect [Zhu et al., 2009]. Furthermore, tp100200 and cec100200 exerted slight negative effects on saikosaponin A and saikosaponin D, respectively. This indicates that different saikosaponins exhibit varying responses to environmental factors. However, since the 100–200 cm soil layer is not the primary zone for root absorption in *B. chinense*, these negative associations may be driven by spatial colinearity or regional background differences. For topographic factors, slope showed a weak negative effect, and elev showed a weak positive effect. These results indicate that topography plays a minor role in explaining saikosaponin variation. The positive association between elevation and saikosaponin D may be related to environmental stressors at higher altitudes, such as lower temperatures and stronger solar radiation, which have been reported to promote secondary metabolite accumulation [Zhao et al., 2017]. Regarding slope, the negative association with total saikosaponin content was statistically marginal (P = 0.044) and might not remain significant after correcting for multiple comparisons. Therefore, its actual influence requires further validation.

### 4.4 Protection and utilization strategies

Based on the comprehensive production zoning results for *B. chinense*, corresponding development strategies are proposed for different production zones.

The high production zone represents the optimal synergy between ecological suitability and medicinal quality of *B. chinense*, and should be prioritized as the core production zone. In the Loess Plateau region (most of Shanxi, northern Shaanxi, southeastern Gansu, etc.), the production zones of *B. chinense* should be planned in conjunction with soil and water conservation projects to reduce soil erosion and stormwater damage to the root system. Leveraging the rich germplasm resources in this region, a seed propagation and breeding base for *B. chinense* should be established in the authentic core production areas, such as Hunyuan (Shanxi) and Longxi (Gansu). In the Northeast Plain region (most of Liaoning, western Jilin, etc.), the advantage of flat land should be fully utilized to explore mechanized cultivation technologies and management systems. Soil health should be maintained through an integrated approach combining straw return, organic fertilization, and crop rotation systems. Straw mulching in winter and soil loosening in spring should be implemented to protect *B. chinense* during overwintering. In the Yanshan-Taihang Mountain region (northern and eastern Hebei, western Beijing), the topographic advantages should be fully exploited by developing graded planting plans according to slope gradient and aspect. For areas prone to waterlogging or intense freeze-thaw cycles, measures such as ridge cultivation combined with drainage ditches and straw mulching should be adopted to protect *B. chinense* from waterlogging and freezing damage.

The medium production zone is a potential development area and should be moderately developed on the basis of ecological carrying capacity assessment to avoid ecological degradation and quality instability caused by blind expansion. In the marginal areas of the Loess Plateau, moderate expansion of cultivation should be promoted on the premise of soil and water conservation, along with the adoption of crop rotation systems and the establishment of ecological monitoring and exit mechanisms. In the North China Plain, carrying capacity assessments should focus on irrigation feasibility, salinity/alkalinity risks, and drainage conditions, and quality control indicators should be established. In the Northeast region, special attention should be paid to freeze-thaw dynamics and soil structure; soil testing should be conducted to dynamically adjust irrigation and fertilization strategies, and overwintering protection should be strengthened. The low production zone belongs to the ecological buffer zone and should prioritize ecological conservation, supplemented by limited utilization. Small-scale demonstrations and trials may be conducted locally, while large-scale reclamation in ecologically fragile areas is prohibited.

### 4.5 Limitations and future outlook

The data sources for spatial quality prediction were heterogeneous, with inconsistencies in growth years, harvest periods, and processing methods across different studies, which may introduce systematic biases. Moreover, we examined only macro-ecological environmental influences on saikosaponin content without accounting for genetic variation or Genotype (G) × Environment (E) interactions. Consequently, environmental factors alone explain only a portion of the observed spatial variation in saikosaponin content, and future studies should incorporate multiple factors—particularly G × E interactions—to enable more comprehensive investigations.

Due to data limitations and the absence of an independent sampling set, we conducted systematic diagnostic analyses to assess the model’s reliability (VIF, Cook’s distance, robust regression, bootstrap; see **Supplementary Tables 7**-**S10**), which confirmed the structural stability of the model: all predictor VIF values were< 5, Cook’s distance was< 1 for 95% of observations, and coefficient signs remained consistent across 1000 bootstrap resamples. This indicates that although the model’s explanatory power is modest, it reliably captures the directional effects of environmental factors, supporting its utility for identifying relative spatial patterns. Therefore, the PCA-MLR-SP framework is positioned as a tool for macro-scale relative estimation, elucidating the relative contributions of environmental factors to spatial variation, rather than for absolute quantification at the micro-scale.

Furthermore, the predicted ecological suitability zones and production zones in southern China remain unvalidated by field surveys. Future studies should ground-truthing in these areas to assess whether the macro-scale patterns identified here hold at the local level, particularly regarding saikosaponin accumulation under southern climatic conditions.

## 5 Conclusions

This study constructed a comprehensive system for *B. chinense* production zoning by integrating ecological niche modeling and spatial prediction. The main conclusions are as follows:

1. The ecological suitability of *B. chinense* was generally higher in the northern regions compared to the southern regions, forming a gradient extending from the northeast to the southwest. The high ecological suitability zone was mainly distributed across the northern North China Plain, eastern Loess Plateau, and southern Northeast China Plain. Precipitation (Bio13, Bio17) and temperature (Bio6, Bio3, Bio2) were identified as the major factor influencing its ecological distribution.
2. The major environmental factors affecting saikosaponin content and the spatial pattern of medicinal quality were analyzed. Climatic factors (Bio15, Bio17) and soil factor (tp05) significantly affected saikosaponin content, while topographic factors exerted only a weak influence. The overall pattern of relative content demonstrated a clear trend of high in the east and north.
3. The comprehensive production zoning results showed that the high production zone was primarily observed in the Loess Plateau, the northern part of the North China Plain, and parts of the Northeast China Plain, covering 1.1 × 10^6^ km^2^, while the medium and low production zones accounted for 0.7 × 10^6^ km^2^ and 0.4 × 10^6^ km^2^, respectively.

## Data Availability

The original contributions presented in the study are included in the article/**Supplementary Material**. Further inquiries can be directed to the corresponding author.
