# Supplementary material for: Using Biomod2 and spatial quality modeling for comprehensive production zoning of Bupleurum chinense DC
Source: Front Plant Sci. 2026 Sep 4;17:1915251. doi: 10.3389/fpls.2026.1915251 (PMC13585929; doi:10.3389/fpls.2026.1915251)
Supplement: Supplementary file 1 [file DataSheet1.docx]

Supplementary Material

# 1 Supplementary Tables

**Supplementary Table 1.** Data-source table.

| Data type | Data source | Selected year | Original spatial resolution | Direct access URL |
| --- | --- | --- | --- | --- |
| Bioclimatic variables  (Bio1-Bio19) | WorldClim | 1970 to 2000 | 1 km | <http://worldclim.org/>  accessed on 6 August 2024 |
| Bioclimatic variables  (srad、vapr) | WorldClim | 1970 to 2000 | 1 km | <http://worldclim.org/>  accessed on 6 August 2024 |
| Soil property data  (clay1, clay2, sand1, sand2) | HWSD | 2023 | 1 km | <https://www.fao.org/soils-portal/>  accessed on 12 March 2024 |
| Soil quality data (sq1-sq7) | HWSD | 2023 | 1 km | <https://www.fao.org/soils-portal/>  accessed on 12 March 2024 |
| Soil data (Soil) | HWSD | 2023 | 1 km | <https://www.fao.org/soils-portal/>  accessed on 12 March 2024 |
| Elevation (elev) | WorldClim | 1970 to 2000 | 1 km | <http://worldclim.org/>  accessed on 6 August 2024 |
| Monthly climatic variables  (tas5-10, pr5-10, SUNP5-10) | National Ecosystem Data Bank | 1991 to 2020 | 1 km | <https://www.scidb.cn/c/o00119/>  accessed on 25 April 2026 |
| Bioclimatic variables  (Bio20–Bio23) | National Ecosystem Data Bank | 1991 to 2020 | 1 km | <https://www.scidb.cn/c/o00119/>  accessed on 25 April 2026 |
| Soil nutrient and physicochemical property data | National Tibetan Plateau Data Center | 2010 to 2018 | 1 km | <http://data.tpdc.ac.cn/>  accessed on 27 April 2026 |

**Supplementary Table 2.** Environmental variables used in the ecological suitability study.

| Category | Code (unit) | Description | Factors used in modeling |
| --- | --- | --- | --- |
| Climatic factors | Bio1(℃) | Annual mean temperature |  |
|  | Bio2(℃) | Mean diurnal temperature range | √ |
|  | Bio3 | Isothermality (Bio2/Bio7) ×100 | √ |
|  | Bio4(℃) | Temperature seasonality |  |
|  | Bio5(℃) | Max temperature of warmest month | √ |
|  | Bio6(℃) | Min temperature of coldest month | √ |
|  | Bio7(℃) | Temperature annual range |  |
|  | Bio8(℃) | Mean temperature of wettest quarter |  |
|  | Bio9(℃) | Mean temperature of driest quarter |  |
|  | Bio10(℃) | Mean temperature of warmest quarter |  |
|  | Bio11(℃) | Mean temperature of coldest quarter |  |
|  | Bio12(mm) | Annual precipitation |  |
|  | Bio13(mm) | Precipitation of wettest month | √ |
|  | Bio14(mm) | Precipitation of driest month |  |
|  | Bio15(%) | Precipitation seasonality | √ |
|  | Bio16(mm) | Precipitation of wettest quarter |  |
|  | Bio17(mm) | Precipitation of driest quarter | √ |
|  | Bio18(mm) | Precipitation of warmest quarter |  |
|  | Bio19(mm) | Precipitation of coldest quarter |  |
|  | srad (kJ·m^-2^·d^-1^) | Solar radiation | √ |
|  | vapr (hPa) | Vapour pressure |  |
| Soil factors | soil | Soil Type | √ |
|  | clay1 | Topsoil Clay Fraction (0～30cm) | √ |
|  | clay2 | Subsoil Clay Fraction (30～100cm) |  |
|  | sand1 | Topsoil Sand Fraction (0～30cm) | √ |
|  | sand2 | Subsoil Sand Fraction (30～100cm) |  |
|  | sq1 | Nutrient availability | √ |
|  | sq2 | Nutrient retention capacity | √ |
|  | sq3 | Rooting conditions | √ |
|  | sq4 | Oxygen availability to roots | √ |
|  | sq5 | Excess salts | √ |
|  | sq6 | Toxicity | √ |
| Topographical factors | elev (m) | Elevation above sea level | √ |
|  | slope (%) | Slope | √ |
|  | aspect | Aspect | √ |

**Supplementary Table 3.** Environmental variables used in the spatial quality study.

| Category | Code (unit) | Description |
| --- | --- | --- |
| Climatic factors | Bio1(℃) | Annual mean temperature |
|  | Bio2(℃) | Mean diurnal temperature range |
|  | Bio3 | Isothermality (Bio2/Bio7) ×100 |
|  | Bio4(℃) | Temperature seasonality |
|  | Bio5(℃) | Max temperature of warmest month |
|  | Bio6(℃) | Min temperature of coldest month |
|  | Bio7(℃) | Temperature annual range |
|  | Bio8(℃) | Mean temperature of wettest quarter |
|  | Bio9(℃) | Mean temperature of driest quarter |
|  | Bio10(℃) | Mean temperature of warmest quarter |
|  | Bio11(℃) | Mean temperature of coldest quarter |
|  | Bio12(mm) | Annual precipitation |
|  | Bio13(mm) | Precipitation of wettest month |
|  | Bio14(mm) | Precipitation of driest month |
|  | Bio15(%) | Precipitation seasonality |
|  | Bio16(mm) | Precipitation of wettest quarter |
|  | Bio17(mm) | Precipitation of driest quarter |
|  | Bio18(mm) | Precipitation of warmest quarter |
|  | Bio19(mm) | Precipitation of coldest quarter |
|  | srad (kJ·m^-2^·d^-1^) | Solar radiation |
|  | vapr (hPa) | Vapour pressure |
|  | tas5-10(℃) | Average temperature during the growth period |
|  | pr5-10(mm) | Average precipitation during the growth period |
|  | SUNP5-10(%) | The average percentage of sunshine during the growth period |
|  | Bio20(℃) | Annual cumulative temperature above 0°C |
|  | Bio21(℃) | Annual cumulative temperature above 5°C |
|  | Bio22(mm) | Annual potential evapotranspiration |
|  | Bio23 | Moisture Index |
| Soil factors | pH | Soil pH |
|  | soc (g/kg) | Soil organic carbon content |
|  | socd (kg/m^2^) | Soil organic carbon density |
|  | cec (cmol(+)/kg) | Cation exchange capacity |
|  | tn (g/kg) | Total nitrogen content |
|  | tnd (kg/m^2^) | Total nitrogen density |
|  | tp (g/kg) | Total phosphorus content |
|  | tpd (kg/m^2^) | Total phosphorus density |
|  | tk (g/kg) | Total potassium content |
|  | tkd (kg/m^2^) | Total potassium density |
|  | bd (g/cm^3^) | Bulk density |
|  | cf (% by volume) | Gravel content |
|  | btsnd (g/kg) | Sand content |
|  | btslt (g/kg) | Silt content |
|  | btcly (g/kg) | Clay content |
|  | thickness (cm) | Soil layer thickness |
|  | texcls | Soil texture class (USDA) |
| Topographical factors | elev (m) | Elevation above sea level |
|  | slope (%) | Slope |
|  | aspect | Aspect |

**Supplementary Table 4.** Pearson correlation coefficients between saikosaponin content and soil factors. (* indicates statistical significance at p < 0.05 level; ** indicates statistical significance at p < 0.01 level.)

| Soil factor | A(r) | D(r) | Total(r) | Soil factor | A(r) | D(r) | Total(r) |
| --- | --- | --- | --- | --- | --- | --- | --- |
| tp05 | 0.222** | 0.328** | 0.312** | ph05 | 0.006 | 0.212* | 0.138 |
| tp515 | 0.14 | 0.300** | 0.257** | ph515 | 0.014 | 0.209* | 0.140 |
| tp1530 | 0.012 | 0.195* | 0.130 | ph1530 | 0.020 | 0.202* | 0.138 |
| tp3060 | 0.042 | 0.180* | 0.134 | ph3060 | 0.045 | 0.199* | 0.148 |
| tp100200 | -0.187* | -0.027 | -0.103 | ph60100 | 0.019 | 0.184* | 0.126 |
| tpd05 | 0.189* | 0.302** | 0.280** | ph100200 | 0.024 | 0.195* | 0.136 |
| tpd515 | 0.152 | 0.299** | 0.261** | cec05 | -0.055 | -0.207* | -0.157 |
| tpd1530 | 0.077 | 0.235** | 0.186* | cec515 | -0.077 | -0.237** | -0.187* |
| tpd3060 | 0.066 | 0.219** | 0.171* | cec1530 | -0.072 | -0.227** | -0.178* |
| tpd60100 | 0.032 | 0.178* | 0.128 | cec3060 | -0.108 | -0.253** | -0.211* |
| tpd0100 | 0.074 | 0.228** | 0.179* | cec60100 | -0.116 | -0.276** | -0.230** |
| tk1530 | 0.024 | 0.178* | 0.125 | cec100200 | -0.090 | -0.285** | -0.223** |
| tk3060 | 0.031 | 0.179* | 0.129 | btslt05 | 0.136 | 0.247** | 0.221** |
| tk60100 | 0.046 | 0.200* | 0.149 | btslt515 | 0.169* | 0.238** | 0.229** |
| tkd05 | 0.048 | 0.198* | 0.149 | btslt1530 | 0.092 | 0.207* | 0.175* |
| tkd515 | 0.077 | 0.226** | 0.180* | btslt3060 | 0.081 | 0.210* | 0.172* |
| tkd1530 | 0.082 | 0.236** | 0.188* | btslt020 | 0.140 | 0.241** | 0.218* |
| tkd3060 | 0.053 | 0.213* | 0.161 | btsnd05 | -0.142 | -0.213* | -0.202* |
| tkd60100 | 0.103 | 0.234** | 0.197* | thickness | 0.173* | 0.243** | 0.235** |
| tkd0100 | 0.081 | 0.229** | 0.184* | btsnd515 | -0.174* | -0.209* | -0.213* |
| cf05 | -0.128 | -0.254** | -0.221** | btsnd1530 | -0.105 | -0.185* | -0.167 |
| cf515 | -0.133 | -0.258** | -0.226** | btsnd3060 | -0.096 | -0.173* | -0.155 |
| cf1530 | -0.134 | -0.243** | -0.217* | btsnd020 | -0.150 | -0.214* | -0.206* |
| cf3060 | -0.103 | -0.224** | -0.191* | tn05 | -0.106 | -0.177* | -0.162 |
| cf60100 | -0.169* | -0.230** | -0.225** | soc05 | -0.110 | -0.170* | -0.159 |

**Supplementary Table 5.** Rotated component loadings for climatic factors.

| Climatic factor | 1 | 2 |
| --- | --- | --- |
| Bio14 | -0.149 | 0.971 |
| Bio15 | 0.672 | -0.448 |
| Bio2 | 0.903 | -0.235 |
| Bio22 | -0.933 | 0.306 |
| Bio7 | 0.968 | -0.146 |
| Bio4 | 0.945 | -0.077 |
| SUNP5_10 | 0.806 | -0.467 |

**Supplementary Table 6.** Rotated component loadings for soil factors.

| Soil factor | 1 | 2 | 3 | 4 |
| --- | --- | --- | --- | --- |
| tpd515 | 0.741 | 0.173 | -0.234 | 0.581 |
| tpd3060 | 0.639 | 0.069 | -0.393 | 0.576 |
| tp05 | 0.425 | 0.293 | -0.167 | 0.785 |
| tp515 | 0.447 | 0.274 | -0.119 | 0.791 |
| tpd0100 | 0.698 | 0.121 | -0.355 | 0.553 |
| tpd1530 | 0.722 | 0.090 | -0.289 | 0.578 |
| tpd05 | 0.715 | 0.162 | -0.268 | 0.587 |
| thickness | 0.555 | 0.399 | -0.449 | 0.297 |
| tkd0100 | 0.875 | 0.289 | -0.217 | 0.134 |
| tkd1530 | 0.889 | 0.231 | -0.240 | 0.113 |
| tkd515 | 0.890 | 0.199 | -0.191 | 0.138 |
| tkd60100 | 0.845 | 0.320 | -0.208 | 0.153 |
| cf3060 | -0.899 | -0.079 | 0.276 | -0.190 |
| cf515 | -0.924 | -0.010 | 0.134 | -0.181 |
| cf60100 | -0.861 | -0.118 | 0.287 | -0.186 |
| cec100200 | -0.322 | -0.018 | 0.845 | -0.049 |
| cec1530 | -0.252 | -0.043 | 0.938 | -0.134 |
| cec3060 | -0.249 | -0.004 | 0.949 | -0.119 |
| cec515 | -0.236 | -0.045 | 0.928 | -0.148 |
| cec60100 | -0.290 | 0.013 | 0.920 | -0.153 |
| cf05 | -0.924 | 0.014 | 0.138 | -0.176 |
| cf1530 | -0.925 | -0.018 | 0.204 | -0.187 |
| btslt515 | 0.088 | 0.973 | -0.048 | 0.106 |
| btsnd020 | -0.170 | -0.959 | -0.024 | -0.081 |
| btsnd05 | -0.162 | -0.961 | -0.065 | -0.029 |
| btsnd515 | -0.087 | -0.975 | -0.090 | -0.034 |
| btslt020 | 0.171 | 0.954 | -0.129 | 0.137 |
| btslt05 | 0.176 | 0.957 | -0.079 | 0.111 |
| btslt1530 | 0.080 | 0.968 | -0.055 | 0.086 |
| btslt3060 | 0.035 | 0.926 | -0.095 | 0.164 |

**Supplementary Table 7.** Variance Inflation Factor (VIF) for regression coefficients.

| Variable | VIF (Original, n=138) | VIF (After outlier removal, n=127) |
| --- | --- | --- |
| Climate_PC1 | 2.38 | 3.54 |
| Climate_PC2 | 1.49 | 1.60 |
| Soil_PC1 | 1.46 | 2.03 |
| Soil_PC2 | 1.62 | 1.94 |
| Soil_PC3 | 1.76 | 2.14 |
| Soil_PC4 | 1.03 | 1.05 |

**Supplementary Table 8.** Coefficient comparison: OLS vs. Robust M-estimation regression.

| Variable | OLS Estimate | Robust Estimate | Sign Consistency |
| --- | --- | --- | --- |
| (Intercept) | 0.6856 | 0.6617 | ✓ |
| Climate_PC1 | −0.0753 | −0.0736 | ✓ (negative) |
| Climate_PC2 | 0.0350 | 0.0421 | ✓ (positive) |
| Soil_PC1 | 0.0553 | 0.0512 | ✓ (positive) |
| Soil_PC2 | −0.0244 | −0.0236 | ✓ (negative) |
| Soil_PC3 | −0.0155 | −0.0054 | ✓ (negative) |
| Soil_PC4 | 0.0258 | 0.0182 | ✓ (positive) |

**Supplementary Table 9.** Coefficient comparison before and after removing influential observations (Cook's Distance > 0.0305).

| Variable | Original (n=138) | After Removal (n=127) | Sign Changed? |
| --- | --- | --- | --- |
| (Intercept) | 0.6856 | 0.6397 | No |
| Climate_PC1 | −0.0753 | −0.0797 | No |
| Climate_PC2 | 0.0350 | 0.0356 | No |
| Soil_PC1 | 0.0553 | 0.0470 | No |
| Soil_PC2 | −0.0244 | −0.0328 | No |
| Soil_PC3 | −0.0155 | −0.0170 | No |
| Soil_PC4 | 0.0258 | 0.0267 | No |

**Supplementary Table 10.** Bootstrap 95% BCa confidence intervals (1,000 resamples)

| Variable | Original Model [2.5%, 97.5%] | Crosses Zero? | After Removal [2.5%, 97.5%] | Crosses Zero? |
| --- | --- | --- | --- | --- |
| (Intercept) | [0.644, 0.740] | No | [0.599, 0.677] | No |
| Climate_PC1 | [−0.182, 0.016] | Yes | [−0.167, −0.003] | No |
| Climate_PC2 | [−0.036, 0.094] | Yes | [−0.021, 0.093] | Yes |
| Soil_PC1 | [−0.007, 0.123] | Yes | [−0.010, 0.109] | Yes |
| Soil_PC2 | [−0.095, 0.041] | Yes | [−0.091, 0.020] | Yes |
| Soil_PC3 | [−0.083, 0.075] | Yes | [−0.076, 0.045] | Yes |
| Soil_PC4 | [−0.026, 0.078] | Yes | [−0.008, 0.072] | Yes |

# 2 Supplementary Figures

**Supplementary Figure 1.** Correlation heatmap of climatic factors.

**Supplementary Figure 2.** Correlation heatmap of topographic factors.

**Supplementary Figure 3.** Comparison of EQI rankings with different ESI-SQI weighting schemes.

# 3 Summary of Research Methods

The ecological suitability research utilized the Biomod2 platform to conduct ecological niche modeling by integrating sample distribution data and environmental variable data. Parameters were configured based on each model's design and research goals to maximize performance. To ensure model robustness, random sampling was applied to create two pseudo-absence datasets for evaluating sampling uncertainty. In the model training stage, data were randomly partitioned into training (80%) and validation (20%) subsets. To assess stability, this entire procedure was independently replicated ten times, allowing for model result verification.This process generated an Ecological Suitability Index (ESI) and delineated ecological suitability zones across various grades. Additionally, the key factors influencing ecological suitability were identified.

The spatial quality was assessed using stepwise regression and the PCA-MLR-SP framework. During this process, the key factors affecting saikosaponin content were studied through correlation analysis. Meanwhile, all climatic and soil variables were subjected to PCA for dimensionality reduction, and the extracted principal component scores were used as independent variables in a MLR model to establish the relationship with saikosaponin content. Subsequently, ArcGIS 10.8 was applied to calculate the Spatial Quality Index (SQI), applying the regression equations to the identified ecological suitability zones for *B. chinense* to derive spatial distribution of relative content. Finally, the Ecological Quality Index (EQI) was derived by integrating the ESI and the SQI, which was then used to generate the comprehensive production zoning for *B. chinense.*
